# Patterning Graphene Film by Magnetic-assisted UV Ozonation

**DOI:** 10.1038/srep46583

**Published:** 2017-04-19

**Authors:** Yixuan Wu, Haihua Tao, Shubin Su, Huan Yue, Hao Li, Ziyu Zhang, Zhenhua Ni, Xianfeng Chen

**Affiliations:** 1 State Key Laboratory of Advanced Optical Communication Systems and Networks, Department of Physics and Astronomy, Shanghai Jiao Tong University, 200240 Shanghai, China; 2 State Key Laboratory of Functional Materials for Informatics, Shanghai Institute of Microsystem and Information Technology, Chinese Academy of Sciences, 200050 Shanghai, China; 3 Department of Physics, Southeast University, 211189 Nanjing, China

## Abstract

Developing an alternative method for fabricating microscale graphene patterns that overcomes the obstacles of organic contamination, linewidth resolution, and substrate damaging is paramount for applications in optoelectronics. Here we propose to pattern chemical vapor deposition grown graphene film through a stencil mask by magnetic-assisted ultraviolet (UV) ozonation under irradiation of a xenon excimer lamp. In this process, the paramagnetic oxygen molecules and photochemically generated oxygen radicals are magnetized and attracted in an inhomogenous external magnetic field. As a consequence, their random motions convert into directional, which can greatly modify or enhance the quality of graphene patterns. Using a ferromagnetic steel mask, an approximately vertical magnetic-field-assisted UV ozonation (B_Z_ = 0.31 T, ∇B_Z_ = 90 T · m^−1^) has a capability of patterning graphene microstructures with a line width of 29 μm and lateral under-oxidation less than 4 μm. Our approach is applicable to patterning graphene field-effect transistor arrays, and it can be a promising solution toward resist-free, substrate non-damaging, and cost effective microscale patterning of graphene film.

Patterning graphene film is a significant step in fabricating graphene-based elements for both fundamental studies and industrial applications^1^,^2^,^3^,^4^,^5^,^6^,^7^,^8^,^9^,^10^,^11^,^12^,^13^,^14^,^15^,^16^. Besides taking direct bottom-up fabrication routes^1^,^2^, various top-down etching solutions are used to cut graphene film into certain patterns^3^,^4^,^5^,^6^,^7^,^8^,^9^,^10^,^11^,^12^,^13^,^14^,^15^,^16^. Electron-beam lithography and photolithography techniques are widely used for their high resolution capability to pattern micro-/nanostructures. However, they both incur resist contamination, and as a consequence inevitably degrade graphene quality after the lithography process^3^,^4^,^5^,^6^,^7^. In order to circumvent this obstacle, direct focused ion beam and laser writing techniques are employed^8^,^9^,^10^,^11^,^12^. As for the focused ion beam, it has a capability of patterning graphene with nanometer scale resolution. However, coexistence of high expense, low productivity, and damage to the supporting substrate limits its applications^8^,^9^. As a contrast, direct laser writing is popularly used to pattern large-area chemical vapor deposition (CVD) grown graphene film due to its high productivity and resist-free characteristics, even though inevitable drawbacks like coarse edges and serious damage to the supporting substrates still exist^10^,^11^,^12^.

In order to overcome these problems in graphene patterning, some alternative solutions have been proposed in the past few years, like TiO_2_-based photocatalysis^13^, resist-free reactive ion etching (RIE) with oxygen and argon plasmas^14^,^15^, and UV ozonation^16^. Weak oxidation of TiO_2_-based photocatalysis and the subsequent complicating disposals prevent its development^13^. As for RIE plasma etching technique, the positively charged ions are electrically accelerated to acquire a directional motion toward substrate, in which way the quality of graphene patterning is improved^17^. Even so, severe lateral under-oxidation up to ten micrometers was induced for graphene underneath the mask due to diffusion of those highly dynamic gaseous reactants^14^,^15^. UV ozonation, a kind of mild oxidation compared with oxygen plasma, showed to be too weak to pattern CVD grown graphene film in the previous study, though it could cut graphene oxide into 2-μm-wide strips when assisted with the ultrasonic wave treatment and high-temperature annealing^16^. Even for a high-temperature enhanced UV ozonation^18^, seeking a solution to making the electrically neutral oxygen radicals move directionally as that of the positively charged ions in RIE process, would be crucial to attain high-quality graphene patterning.

In this study, we propose to pattern CVD grown graphene film through stencil masks by magnetic-assisted UV ozonation. An external inhomogenous magnetic field can magnetize the paramagnetic oxygen molecules and photochemically dissociated oxygen radicals, and induce strong attractive magnetic forces^19^,^20^,^21^,^22^,^23^,^24^. As a consequence, random motions of the oxygen molecules and radicals turn into directional, and it can tremendously enhance the quality of graphene patterning when a vertical magnetic field (relative to graphene film) is applied. This magnetic-induced directional and enhanced photochemical reaction is consistent with the report on macroscopic deflection of the flow of oxygen gas when they were put in an inhomogeneous magnetic field (B = 1 T, ∇B = 100 T · m^−1^)^20^,^21^. Using a ferromagnetic steel mask, the vertical magnetic-field-assisted UV ozonation (B_*Z*_ = 0.31 T, ∇B_*Z*_ = 90 T · m^−1^) has a capability of patterning 29-μm-wide conformal graphene microstructures with the lateral under-oxidation less than four micrometers, and this approach is applicable to patterning graphene field-effect transistor (FET) arrays.

## Results

### Strategy for patterning graphene by magnetic-assisted UV ozonation

A home-designed UV ozonation vacuum machine is used for graphene patterning with a xenon excimer lamp installed on top inside the chamber (Supplementary Figure 1). The electric power supplied for the UV source is fixed at 1.5 kW. The distance between the UV lamp and graphene film (i.e., working distance) is fixed at 20 mm. To acquire a strong photochemical oxidation, the chamber is filled with a small fraction (10 Pa) of oxygen gas in a nitrogen (N_2_) atmosphere with the total pressure of 1 atm. A cube permanent magnet is placed underneath the SiO_2_/Si substrate in order to exert a vertical inhomogenous magnetic field (Supplementary Figure 2a).

Graphene patterns are formed through different stencil masks at room temperature after four cycles of consecutive UV ozonation treatments (10 min/cycle, with the same initial oxidation parameters). Nonmagnetic sapphire mask, and magnetic masks made of nickel grid (the commercial 400 mesh for transmission electron microscope) and molybdenum doped steel grid are used individually for patterning graphene film. The magnetization of nickel mask is along its surface when put in a magnetic field, and as a result it cannot be used to pattern graphene film in a vertical magnetic-field-assisted UV ozonation (see Supplementary Figure 2a). However, an approximately horizontal magnetic field provided by a stack of bar magnets can make it stable (see Supplementary Figure 2b). The steel mask is magnetized along a direction perpendicular to its surface. As a consequence, its contact with graphene film can be improved due to strong magnetic attraction close to one pole of the cube magnet (see Supplementary Figure 2c).

### Working mechanism of magnetic-assisted UV ozonation

**Figure 1:**
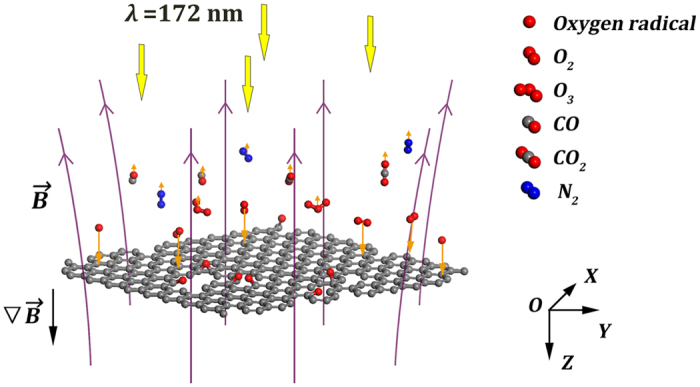
Schematic illustration of the magnetic-induced directional motion for gaseous substances in UV ozonation. Yellow arrows represent UV irradiation at λ = 172 nm; Purple arrows represent the magnetic field 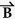; Orange arrows represent the magnetic force 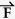 for paramagnetic (O, O_2_) and diamagnetic (O_3_, CO, CO_2_, N_2_) substances.

UV ozonation is a type of photochemical oxidation characterized by random etching of the oxidative reactants^16^,^18^,^25^,^26^,^27^,^28^. As schematically shown in Fig. 1, oxygen molecules are primarily dissociated to ground-state O(^3^P) atoms, or called oxygen radicals under irradiation of the xenon excimer lamp centered at 172 nm. Then, these oxygen radicals oxidize graphene into carbon monoxide (CO) and carbon dioxide (CO_2_) molecules, or they combine with oxygen molecules to form instable ozone (O_3_). As confirmed by the X-ray photoelectron spectroscopy analyses (Supplementary Figure 3 and Supplementary Note 1), no extra contamination is introduced to graphene except for the adsorbed carbonyl (-C=O) and epoxide (C-O) oxygen functional groups^18^,^28^.

In the UV ozonation process, oxygen molecule behaves as a strong paramagnetic substance, while the reactive products (O_3_, CO and CO_2_ molecules) and the prefilled gaseous nitrogen behave as weak diamagnetic substances^29^,^30^,^31^,^32^. The magnetization capability of all these gases is characterized by the molar magnetic susceptibility (20 °C, 1 atm) as listed in Table 1, where we can see that the magnetic susceptibility of oxygen molecule is two orders higher than those of the diamagnetic molecule gases^29^,^30^,^31^,^32^. For comparison, the referred volume magnetic susceptibility for oxygen and nitrogen molecules are converted into molar susceptibility as elaborated in detail in Supplementary Note 2^23^,^29^.

When an inhomogenous magnetic field (B_*Z*_ = 0.31 T, ∇B_*Z*_ = 90 T · m^−1^) is applied to UV ozonation, the paramagnetic oxygen molecule and radical, which have the magnetic moment μ_B_ equal to 2.0 and 1.67 Bohr magnetons, respectively, are magnetized as shown in Fig. 1^23^,^24^. The magnetic forces F_Z_ exerted on one oxygen molecule and radical is deduced to be both in the order of 10^−22^ N toward graphene^24^ from the formula F_Z_ = g_J_μ_B_∇B_Z_, where g_J_ is the Landé g-factor taking values between 1 and 2. The paramagnetic-induced attractive magnetic force converts the random motions of oxygen molecules and radicals into directional ones as demonstrated by the longer orange arrows pointing to graphene film. On the contrary, all the diamagnetic molecules (N_2_, O_3_, CO_2_, CO) experience weak repulsive magnetic forces away from graphene surface due to their two-order smaller negative magnetic susceptibilities^21^,^29^,^30^,^31^,^32^, which may further facilitate ongoing of the photochemical reaction. As a consequence, UV ozonation turns into directional, and in principle it can improve the quality of graphene patterning.

In the above discussion, we have simplified the theoretical model by assuming that all these gaseous substances work at 1 atm at room temperature in the magnetic-assisted UV ozonation. As a matter of fact, except for nitrogen gas, all other gaseous substances, including the paramagnetic oxygen molecules and radicals (10 Pa or less), have much lower partial pressures. As a consequence, it may further enhance directionality due to decreasing probability of intermolecular collisions adjacent to graphene surface in UV ozonation.

### Patterning graphene through a nickel mask by UV ozonation

**Figure 2:**
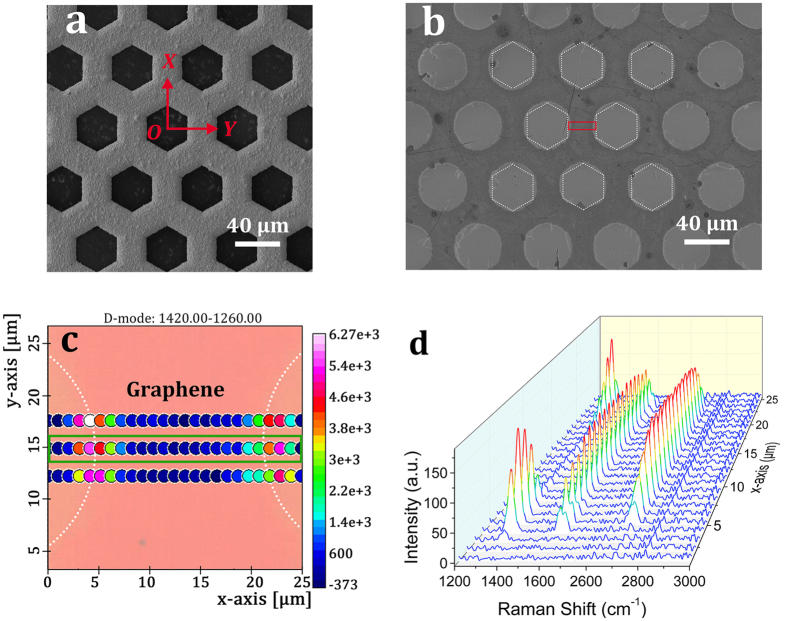
Patterning graphene through a nickel mask by UV ozonation. SEM topographical images of (**a**) the nickel mask and (**b**) graphene microstructures patterned by UV ozonation without any magnetic assistance. White dotted hexagons in (**b**) represent actual hole positions of the mask. (**c**) Micro-Raman map of the defect band (D band) intensity in the region denoted by the red rectangle in (**b)**, and (**d**) its Raman spectrum evolution for the dots outlined by the green rectangle in (**c**). a.u., arbitrary unit; graphene edges are all denoted by white dotted lines in Raman mapping.

UV ozonation is used to pattern graphene film through a ten-micrometer-thick nickel mask without applying any magnetic field. The mask has a honeycomb structure of hexagonal holes with the lattice constant of 62 μm and rib width of 26 μm as shown in the SEM image of Fig. 2a. The patterned holes deform into circular and appear over-etched, especially in the region away from corners when compared with the mask profile as partially outlined by the white hexagons in Fig. 2b. The minimum rib width connecting two adjacent holes decreases to 21 ± 1 μm, a few micrometers narrower than that of the mask. Raman spectroscopy is used to evaluate the quality of graphene microstructures^14^,^33^. Figure 2c shows the defect band (D band) map in the region denoted by the red rectangle in Fig. 2b. From the color variation, we can see the D band shows up in the region along graphene edges. The corresponding Raman spectrum evolution for the green-outlined dots (Fig. 2d) shows that the lateral under-oxidation across the graphene edges is 4–5 μm. Herein, we use a mechanically exfoliated high-quality monolayer graphene, which is testified to be free of defect bands in the edge area, as reference (see Supplementary Figure 4).

A horizontal magnetic-field-assisted UV ozonation (B_Y_ = 40 mT, ∇B_OY_ = 2 T · m^−1^) can completely modify the graphene pattern even though the same nickel mask is used (see Supplementary Figure 5 and Supplementary Note 3 for more information). The results indicate that the paramagnetic oxygen radicals and molecules turn into directional motions in the horizontal magnetic field, and as a result it makes the photochemical oxidation directional. In spite of the unwanted lateral under-oxidation, a properly designed and well controlled magnetic field may provide a solution to intentionally modifying graphene patterns.

### Patterning graphene through a sapphire mask by a vertical magnetic-field-assisted UV ozonation

**Figure 3:**
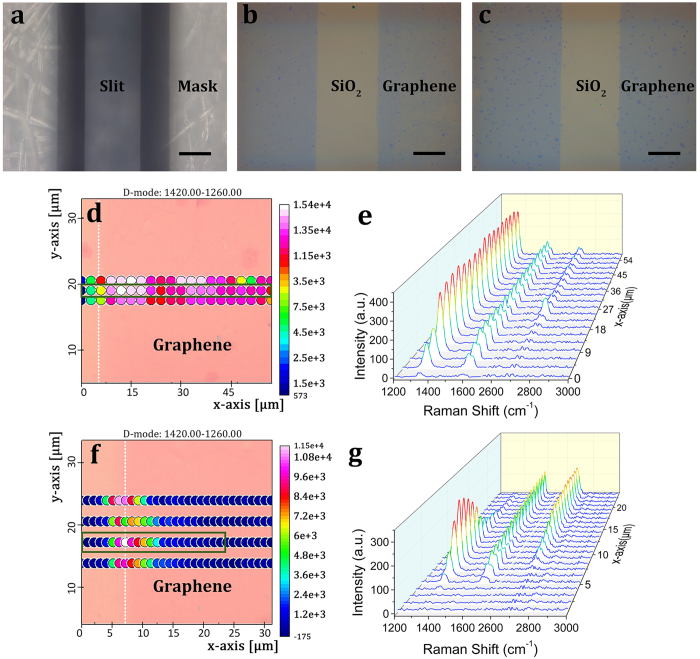
Comparison of graphene patterning through a synthetic sapphire mask without and with assistance of an inhomogenous vertical magnetic field in UV ozonation. Optical topographical images of (**a**) the sapphire mask, and graphene patterned (**b**) without and (**c**) with assistance of the vertical magnetic field, respectively. Micro-Raman map of (**d**) the D band intensity crossing the edge of patterned graphene without magnetic assistance and (**e**) the corresponding Raman spectrum evolution for the dots outlined by the green rectangle in (**d**). (**f**) Micro-Raman map of the D band crossing the edge of patterned graphene with assistance of the vertical magnetic field and (**g**) the corresponding Raman spectrum evolution for the dots outlined in the green rectangle in (**f**). All scale bars are 100 μm.

Using a non-magnetic 316-μm-thick sapphire mask shown in Fig. 3a, a vertical magnetic-field-induced directional photochemical reaction (B_*Z*_ = 0.31 T, ∇B_*Z*_ = 90 T · m^−1^) can be intuitively demonstrated when patterning graphene by UV ozonation. Comparing the optical images in Fig. 3b and c, we can see that most of the multilayer graphene nucleuses disappear in the structure patterned without assistance of the vertical magnetic field. Further micro-Raman maps of the D band in Fig. 3d and its corresponding Raman spectrum evolution Fig. 3e indicate that the nonmagnetic-assisted UV ozonation induces severe lateral under-oxidation throughout the graphene pattern. This phenomenon stems from UV penetration through the transparent sapphire mask and the subsequent photochemical reaction propelled by diffusion of ozone, oxygen radicals and molecules. When the vertical magnetic field is applied, the lateral under-oxidation decreases to 11 μm as indicated by Raman spectrum characterizations in Fig. 3f and g. For the nonmagnetic sapphire mask, its contact with graphene is independent of the vertical magnetic field. Therefore, it is the magnetic field that induces the directional motions of oxygen molecules and radicals toward graphene film, and then reduces their lateral diffusion underneath the mask^19^,^21^.

### Patterning graphene through a steel mask by a vertical magnetic-field-assisted UV ozonation

**Figure 4:**
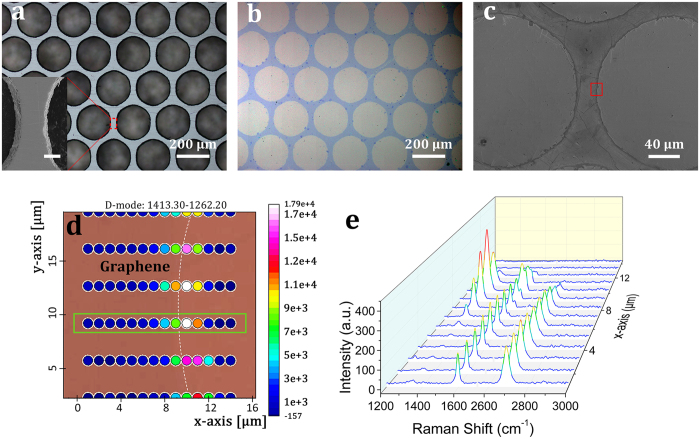
Patterning graphene with a steel mask by applying an inhomogenous vertical magnetic field in UV ozonation. (**a**) Optical microscope image and zoom-in scanning electron micrograph (inset) of the steel mask. The scale bar in the inset is 20 μm. (**b**) Optical microscope image of a graphene pattern, and (**c**) its high-resolution SEM image. (**d**) Micro-Raman map of the D band intensity in the region as denoted in (**c)**. (**e**) Evolution of Raman spectra for the dots outlined by the green rectangle in (**d)**.

Using a magnetic steel mask, the quality of graphene patterning can be improved in the vertical magnetic-field-assisted UV ozonation (B_*Z*_ = 0.31 T, ∇B_*Z*_ = 90 T · m^−1^). Figure 4a shows the optical image of a steel mask composed of a hexagonal lattice of holes with the constant of 220 μm and rib width (at surface) of 29 ± 2 μm. As indicated by the high-resolution SEM image in the inset, these holes have rough sidewalls with protrusions fluctuating within four micrometers. The etched graphene pattern conforms well to the mask profile as shown by the optical image in Fig. 4b. Further high-resolution SEM imaging (Fig. 4c) indicates that there still exist some bright micro/nanofilaments along edges, similar to but sparsely distributed compared to those in Fig. 2b. These residues originate from wrinkles formed during CVD growth and the following transfer of graphene film (see Supplementary Figure 6 for more information)^34^,^35^. Analyses of the Raman map of D band (Fig. 4d) and the corresponding Raman spectrum evolution (Fig. 4e) indicate that the lateral under-oxidation aroused by dissipation of oxygen radicals underneath the mask is 3–4 μm, decreased compared to that using a sapphire mask. The gas-diffusion induced graphene oxidation could be related to a combination of the non-ideal vertical distribution of the magnetic field and the imperfect contact between the mask and graphene.

When a weak magnetic field (B_Z_ = 19 mT, ∇B_Z_ = 4.5 T/m) is applied, the quality of patterned graphene microstructure deteriorates rapidly after the same UV ozonation treatment (see Supplementary Figure 6). Further, when no magnetic field is applied, such pattern cannot form due to severe diffusion and dissipation of oxidative reactants underneath the mask.

The success of graphene patterning by magnetic-assisted UV ozonation is attributed to two major factors. Firstly, the directional motion of oxygen molecules and radicals toward graphene surface can enhance directionality of the photochemical etching process. Secondly, magnetic-induced contact improvement between the stencil mask and graphene film is critical for attaining high quality graphene patterning. Besides the directional oxygen radicals and molecules, the photo-generated weak diamagnetic ozone molecules, which is instable and subject to decomposing into oxygen radicals and molecules, may diffuse underneath the mask and then contribute to the lateral under-oxidation. This opinion is supported by the feasibility of patterning graphene film through a high-quality nickel mask in Fig. 2 when no magnetic field is applied.

Table 2 summaries the traits of patterning graphene film by UV/ozonation when different masks and magnetic fields are used. As can be seen, the best graphene patterning can be obtained when a magnetic steel mask is used in the strong vertical magnetic-field-assisted UV ozonation.

### Patterning graphene FET arrays by the vertical magnetic-field-assisted UV ozonation

**Figure 5:**
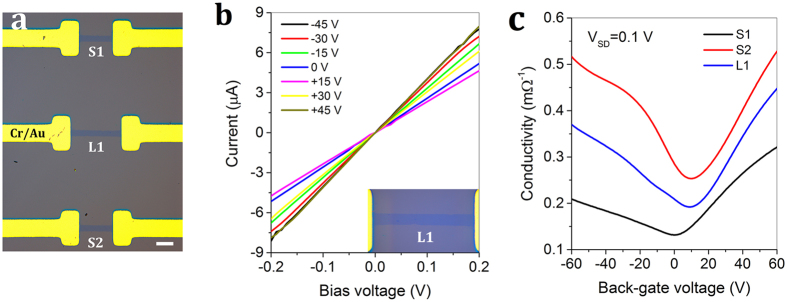
Characterization of the graphene FET array patterned by the vertical magnetic-field-assisted UV ozonation. (**a**) Optical image of the graphene FET array consisting of three devices with two different channel lengths. Scale bar, 200 μm. (**b**) Linear relationship between current and bias voltage for L1 at different back-gate biases ranging from −45 to +45 V in a step of 15 V. The inset shows a zoom-in optical image. (**c**) Conductivity curve as a function of gate bias for the graphene FET array at a fixed source-drain voltage V_SD_ = 0.1 V.

The capability of patterning highly improved graphene microstructures for the vertical magnetic-field-assisted UV ozonation (B_*Z*_ = 0.31 T, ∇B_*Z*_ = 90 T · m^−1^) allows it to fabricate graphene electronic circuits (see Method and Supplementary Figure 7). Figure 5a demonstrates a graphene FET array that consists of three devices with the same channel width of 70 μm and two different channel lengths of 390 μm (named as S1 and S2) and 586 μm (named as L1). The contacts are made of Cr/Au film (5/90 nm thick). Consistent with that in Fig. 4, the lateral under-oxidation remains 3–4 μm as indicated by micro-Raman measurement (see Supplementary Figure 8). Before electrical measurement, the neutral (Dirac) points are shifted close to zero in a high vacuum of 4.5 × 10^−4^ Pa under *in situ* illumination at 254 nm from a low-pressure mercury lamp in a double-chamber UV ozonation machine^18^,^36^. The relationship between source-drain current and the applied bias voltage V_SD_ is linear for all graphene FET elements at different back-gate biases ranging from −45 to +45 V. Figure 5b demonstrates the linear dependence for L1 device, which confirms the ohmic contact between Cr/Au electrodes and the underneath graphene film.

At a fixed source-drain voltage (V_SD_ = 0.1 V), the conductivity curves varying with the gate bias for these three FETs are shown in Fig. 5c. The transfer length method (TLM), which is used for attaining accurate density dependent mobility and contact resistance at relatively high carrier density, is not applicable to the hundreds-of-micrometers-long non-uniform CVD graphene devices^37^. Using a fitting method proposed by Kim, we obtain the highest hole and electron mobilities of ~1682 cm^2^ · V^−1^ · s^−1^ and ~1316 cm^2^ · V^−1^ · s^−1^, respectively^1^,^2^, for the S2 graphene FET device. For the other S1 and L1 devices, the conduction decreases and their hole mobilities are lower than electron mobilities^33^,^38^. Our extra measurements further confirm that the magnetic-assisted UV ozonation is not the only element that determines the conductivity and electron-hole asymmetry conduction. A combination of the neutrality point misalignment caused by non-uniformity due to randomly distributed wrinkles, cracks, multilayer nucleuses, and contamination in the CVD grown graphene film can all contribute to such variation for each individual FET device^34^,^35^,^39^,^40^,^41^.

When lacking a vertical magnetic field, the above discussed 70-μm-wide graphene FET array cannot be successfully patterned by UV ozonation due to severe dissipation of oxidative reactants underneath the mask. Instead, using a 168-μm-wide steel mask, we can only obtain 129-μm-wide channels (Supplementary Figure 9a). Meanwhile, the lateral under-oxidation across graphene channel reaches 40 μm (Supplementary Figure 9b). Further electrical measurements (Supplementary Figure 9c,d) show that the electrical current varies linearly with the source-drain voltage under different back-gate biases (from −60 V to 60 V), and that its conductivity degrades compared to those of the FET devices in Fig. 5c. This electrical deterioration mainly stems from severe lateral under-oxidation across channels by the randomly moving oxidative reactants when lacking a vertical magnetic field.

## Discussion

Compared with the laser writing or reactive ion etching (RIE), the magnetic-assisted UV ozonation has the following characteristics^10^,^11^,^12^,^14^,^15^. Firstly, the unique directional photochemical etching mechanism explains the feasibility of highly improved graphene patterning by the vertical magnetic-field-assisted UV ozonation. Meanwhile, no observable damage is induced to the substrate in the photochemical process. Increasing the magnetic field and its gradient can further enhance the dynamic energies of oxygen radicals and molecules, and its impact on the substrate and the quality of patterned graphene needs to be explored. Secondly, unlike the direct laser writing, its etching productivity does not depend on the area that needs to be etched off since the patterning is a photochemical reaction. Thirdly, it can be applicable to patterning high-quality large-area graphene film provided that the most critical factor, the magnetic field, can be scaled up and well controlled. As known, the other two critical factors, the stencil mask and the fourteen-inch-long xenon excimer lamp, can be readily scaled up.

The magnetic-assisted UV ozonation approach manifests good sample-to-sample consistency and reproducibility for patterning graphene microstrustures. For seeking applications in the field of nanotechnology, it is important to explore the minimum line width that UV ozonation can realize to pattern graphene film. Put aside the quality of graphene film, a high-quality magnetic mask etched with micro/nano-structures may provide a solution to further improving graphene patterning. Besides, a precisely designed and controlled external magnetic field would facilitate improving (such as eliminating wrinkle-incurred residues and diffusion-induced lateral under-oxidation) or intentionally modifying graphene patterns.

## Summary

In summary, we have proposed and demonstrated a new strategy to pattern CVD grown graphene film by magnetic-assisted UV ozonation. By virtue of the paramagnetic property of oxygen molecules/radicals, we can pattern 29-μm-wide graphene microstructure with the lateral under-oxidation less than four micrometers under irradiation of a xenon excimer lamp. The vertical magnetic-field-assisted UV ozonation approach is applicable to patterning graphene FET arrays, and it provides a resist-free, substrate non-damaging, and cost-effective solution to microscale graphene patterning.

## Methods

### Preparation of CVD graphene film on SiO_2_/Si substrate

A “PMMA-mediated” approach was used to transfer CVD grown monolayer graphene on a copper foil onto the thermally grown SiO_2_ film (300 nm) on a highly doped p-type silicon substrate (0.001~0.004 ohm · cm) as follows^33^,^39^,^40^. Firstly, a 200 nm thick PMMA 950 A5 layer was spin-coated on the graphene/copper substrate and then baked for 2 min at 160 °C. Secondly, we removed the copper foil in an etchant of 0.5 M FeCl_3_ aqueous solution after 3 h immersion and then obtained the PMMA/graphene stack layer. Thirdly, the stack was etched by dipping in H_2_O/H_2_O_2_/HCl (20:1:1) and H_2_O/H_2_O_2_/NH_4_OH (20:1:1) solutions successively to remove possible metal residues. After each etching, it was rinsed sufficiently by deionized water and then scooped out onto a clean SiO_2_/Si substrate. Monolayer graphene, predominantly monolayer with randomly distributed multilayer flakes less than 5%, was finally obtained by solving the PMMA in acetone. In order to remove possible organic residues and enhance its contact with the SiO_2_/Si substrate, an extra disposal of annealing in a flow of gas mixture (Ar:H_2_ = 200 sccm:100 sccm) at 290 °C was carried out for three hours.

### Characterizations and electronic measurements

An optical microscope (Leica DM 4000) was used for morphology imaging of the patterned graphene microstructures. A scanning electron microscope (SEM, Zeiss Ultra Plus) under 5 kV and 3 kV biases was used to obtain highly resolved topographical images of masks and graphene patterns, respectively. A confocal micro-Raman spectroscopy (Senterra R200-L) was used to map graphene patterns under excitation of 532 nm laser (50X objective, ~1.2-μm spot size) with the scanning step size of 1 μm. Relative to the sample positioning platform, there exists a shift of ~3-μm upward and ~0.5-μm rightward for the laser positioning system. The *ex situ* XPS spectra were collected using a Kratos Axis Ultra^DLD^ spectrometer (equipped with a monochromatic Al Kα X-ray source) with the anode power of 150 W. A gaussmeter was used to measure the strength and direction of a magnetic field adjacent to graphene surface. Its gradient was approximated by the ratio of the magnetic difference to a certain distance within one millimeter. All the electrical measurements were carried out in a high vacuum chamber (4.5 × 10^−4^ Pa) with a combination of Keithley 6430 and 2400 systems.

## Additional Information

**How to cite this article:** Wu, Y. *et al*. Patterning Graphene Film by Magnetic-assisted UV Ozonation. *Sci. Rep.* **7**, 46583; doi: 10.1038/srep46583 (2017).

**Publisher's note:** Springer Nature remains neutral with regard to jurisdictional claims in published maps and institutional affiliations.

## Supplementary Material

Supplementary Information

## Figures and Tables

**Table 1:** Molar magnetic susceptibility of the paramagnetic and diamagnetic gases at 20 °C and 1 atm.

Gas	O_2_	N_2_	O_3_	CO	CO_2_
Molar magnetic susceptibility (χ_m_: 10^−6^ cm^3^ · mol^−1^)	+3490	−11.8	−18.0	−15.6	−18.7

**Table 2:** Comparison of graphene patterning obtained individually through three types of stencil masks by different magnetic-assisted UV/ozonation processes.

Mask type/Thickness	Nickel/10 μm	Sapphire/316 μm		Steel/30 μm	
Magnetic field	0	0	B_Z_ = 0.31 T, ∇B_Z_ = 90 T/m	B_Z_ = 19 mT, ∇B_Z_ = 4.5 T/m	B_Z_ = 0.31 T, ∇B_Z_ = 90 T/m
Lateral under-oxidation/μm	4–5	>200	8	>30	3–4
Trait	Poor conformity	Magnetic-induced directional etching		Magnetic-enhanced conformity	
