# Supplementary material for: Patterning Graphene Film by Magnetic-assisted UV Ozonation
Source: Sci Rep. 2017 Apr 19;7:46583. doi: 10.1038/srep46583 (PMC5395948; doi:10.1038/srep46583)
Supplement: Supplementary Information [file srep46583-s1.pdf]

Supplementary Information for

Patterning Graphene Film by Magnetic-assisted UV Ozonation

Yixuan Wu[1], Haihua Tao[1,3,*], Shubin Su[1], Huan Yue[1], Hao Li[1], Ziyu Zhang[1], Zhenhua Ni[2], Xianfeng Chen[1,*]

[1] *State Key Laboratory of Advanced Optical Communication Systems and Networks, Department of Physics and Astronomy, Shanghai Jiao Tong University, 200240 Shanghai, China.*

[2] *Department of Physics, Southeast University, 211189 Nanjing, China.*

[3] *State Key Laboratory of Functional Materials for Informatics, Shanghai Institute of Microsystem and Information Technology, Chinese Academy of Sciences, 200050 Shanghai, China.*

Correspondence and requests for materials should be addressed to H.T. (email: tao.haihua@sjtu.edu.cn) or to X.C. (email: xfchen@sjtu.edu.cn)

Supplementary Figures

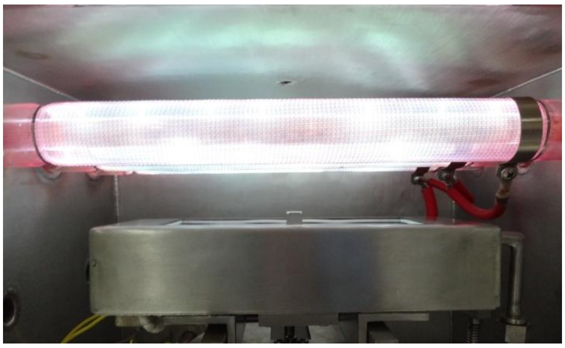

Supplementary Figure 1: A picture of the inner chamber of our home-designed UV ozonation vacuum machine installed with a xenon excimer lamp.

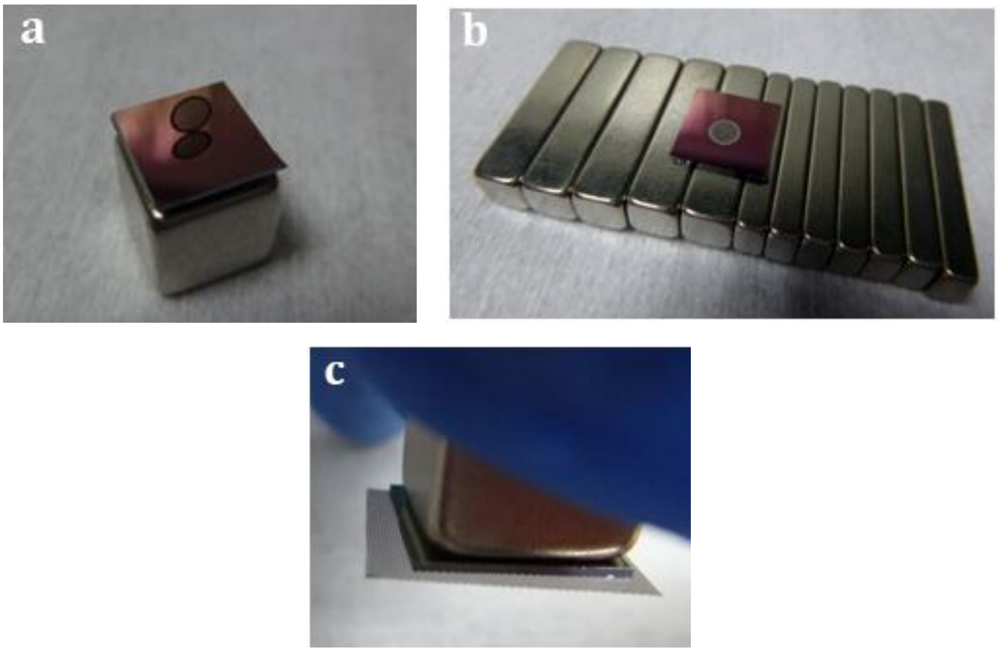

Supplementary Figure 2: Different magnetization between the nickel and steel ferromagnetic masks in an external magnetic field. a) Nickel mask stands up on the substrate when got close to one pole of the magnet due to its magnetization in a direction along the grid surface. **b)** A stack of bar magnets are used to generate an approximately horizontal magnetic field which makes the nickel mask stably lie down on the substrate. The horizontal magnetic field and its gradient are measured to be $40\,\mathrm{mT}$ and $2\,\mathrm{T\cdot m^{-1}}$, respectively, on graphene surface along the piling direction. **c)** Steel mask is attracted to one pole of the magnet upward with the substrate sandwiched between them. The approximately vertical magnetic field and its gradient are measured to be $0.31\,\mathrm{T}$ and $90\,\mathrm{T\cdot m^{-1}}$, respectively, on graphene surface.

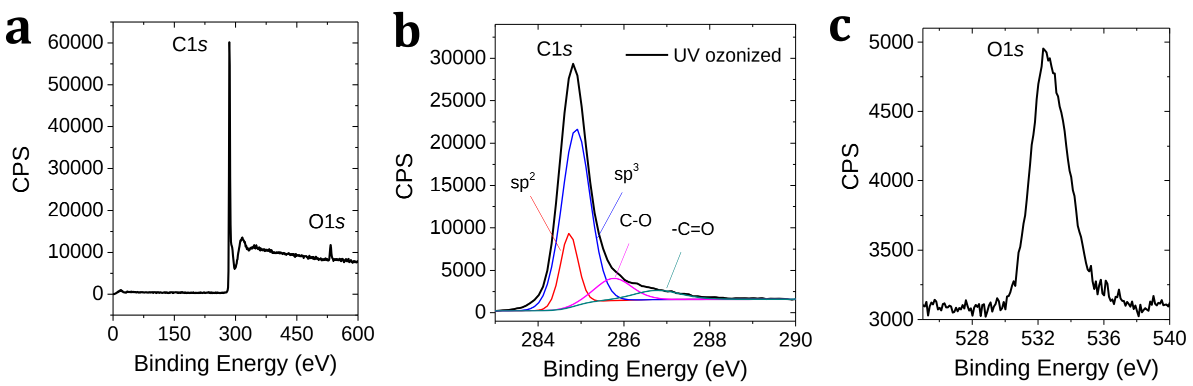

Supplementary Figure 3: XPS spectra for UV ozonized Kish graphite under irradiation of the xenon excimer lamp. a) XPS survey spectrum. Highly resolved narrow-band **b)** C1*s* spectrum fitted with four mixed Gaussian-Lorentzian curves, and **c)** O1*s* spectrum. CPS stands for count per second.

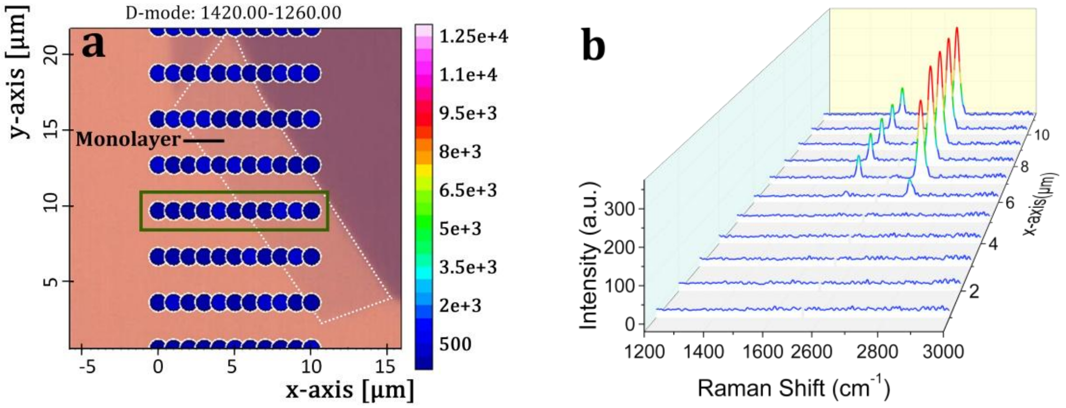

Supplementary Figure 4: Micro-Raman characterization of the exfoliated monolayer graphene flake (outlined by the white dots). a) Raman map of the D band crossing a monolayer graphene, and **b)** the corresponding Raman spectrum evolution along the dots outlined by the green rectangle. a.u., arbitrary unit; graphene edges are all denoted by white dotted lines in Raman mapping. Raman analyses indicate that there is no observable D mode in the edge of exfoliated graphene. Therefore, the map of D mode for graphene patterns can be used to characterize lateral under-oxidation in the UV ozonation.

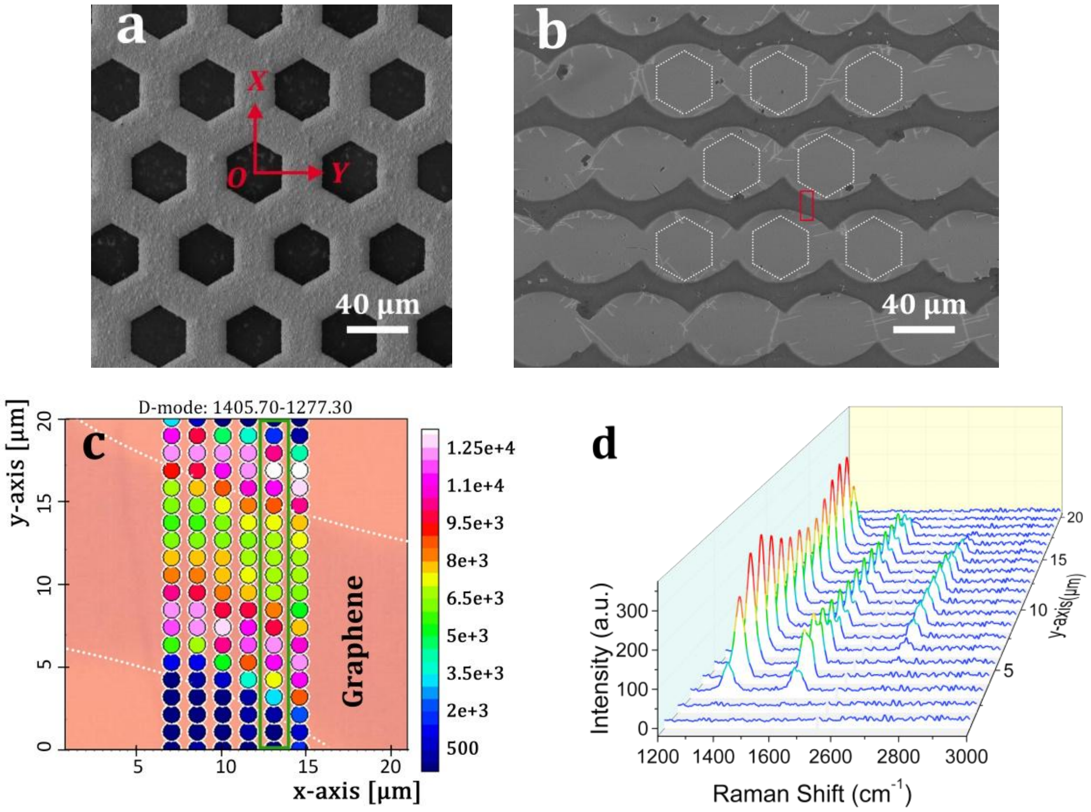

Supplementary Figure 5: Patterning graphene through a nickel mask by horizontal magnetic-field-assisted UV ozonation. SEM topographical images of **a)** the mask and **b)** patterned graphene microstructures with the white dotted hexagons representing the actual hole position of the mask. **c)** Micro-Raman map of D band intensity in the region denoted by the red rectangle in **b**, and **d)** its Raman spectrum evolution for the dots outlined by the green rectangle in **c**.

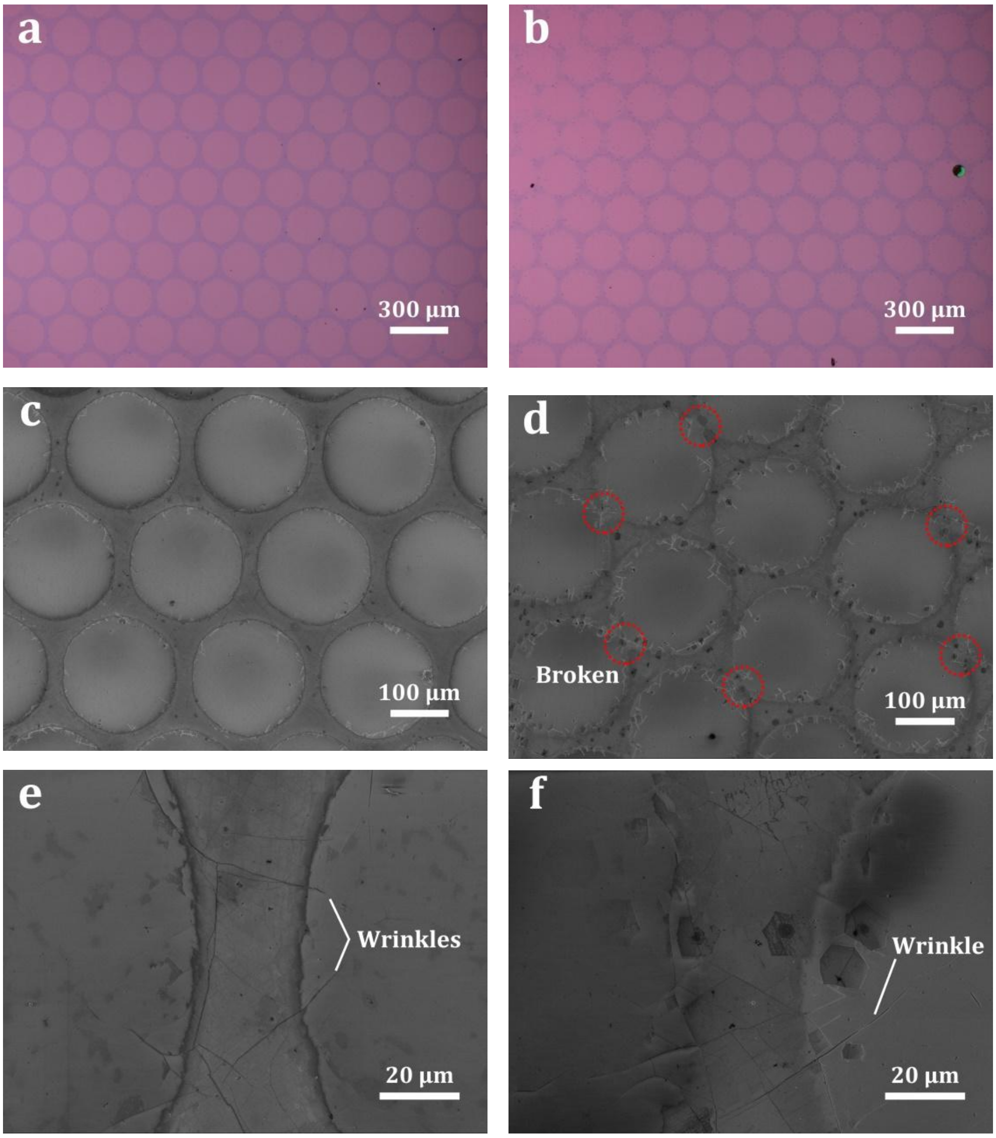

Supplementary Figure 6: Comparison of graphene microstructures patterned through a steel mask by UV ozonation assisted by the strong ($\mathbf{B_Z = 0.31\ T,\ \nabla B_Z = 90\ T/m}$) and weak ($\mathbf{B_Z = 19\ mT,\ \nabla B_Z = 4.5\ T/m}$) vertical magnetic fields. Optical microscope images of the graphene microstructures patterned with assistance of **a)** the strong and **b)** weak magnetic field. High-resolution SEM images of the graphene microstructures patterned with assistance of **c), e)** the strong and **d), f)** weak magnetic field. As indicated, the residual sub-micron filaments originate from wrinkles.

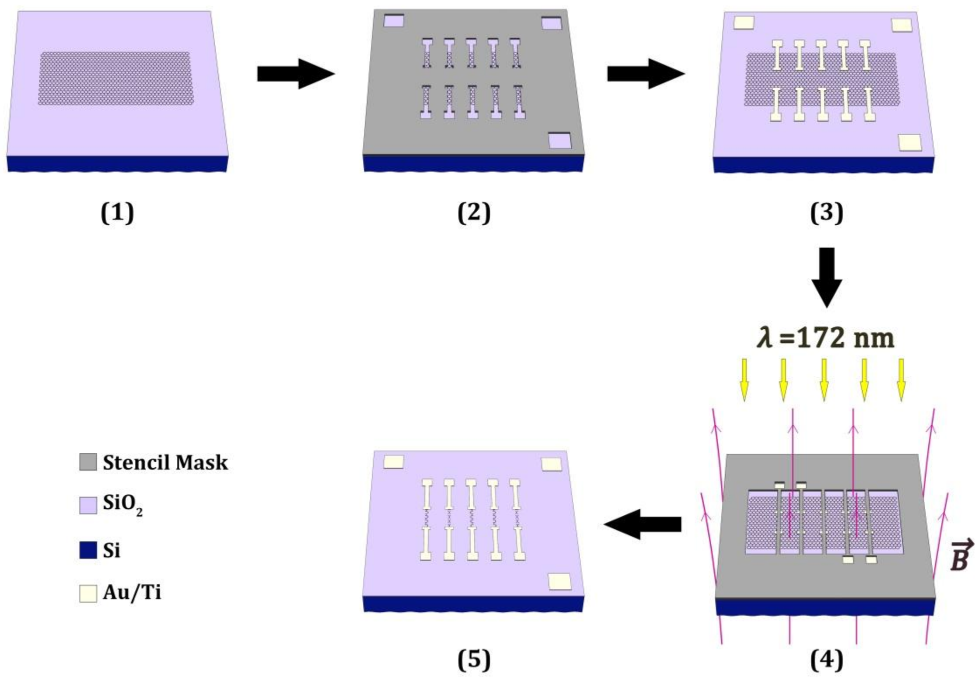

Supplementary Figure 7: Schematic illustration of patterning graphene field-effect transistor (FET) arrays by an inhomogenous vertical-magnetic-field-assisted UV ozonation. (1) Following the transfer of CVD grown graphene onto SiO_2/Si substrate, **(2), (3)** Cr/Au (5/90 nm thick) source-drain electrical contacts are thermally evaporated using a stencil steel mask. **(4)** Then graphene with the metal contacts are aligned with another different steel mask through four windows and etched in the magnetic-assisted UV ozonation machine. **(5)** Graphene FET arrays are finally obtained after etching off the unmasked regions.

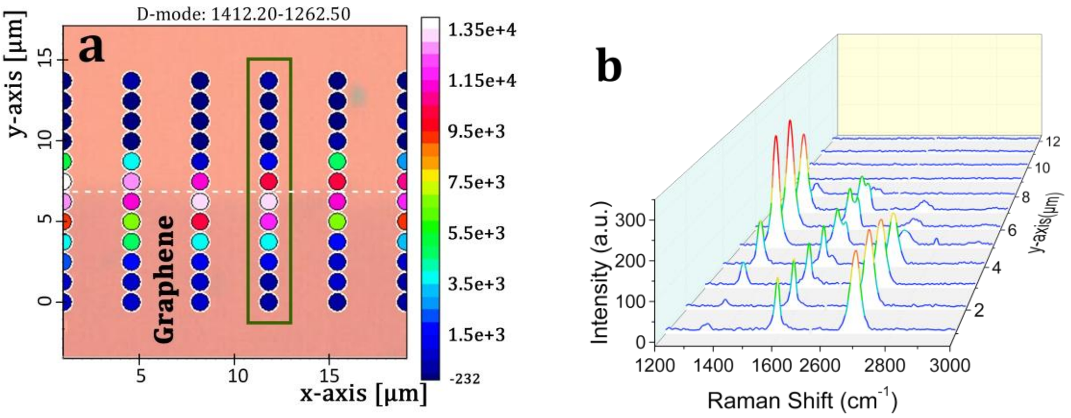

Supplementary Figure 8: Micro-Raman characterization of a graphene FET element patterned by the vertical-magnetic-field-assisted UV ozonation. a) Raman map of the D band crossing one edge of the channel, and **b)** the corresponding Raman spectrum evolution along the dots outlined by the green rectangle in **a**.

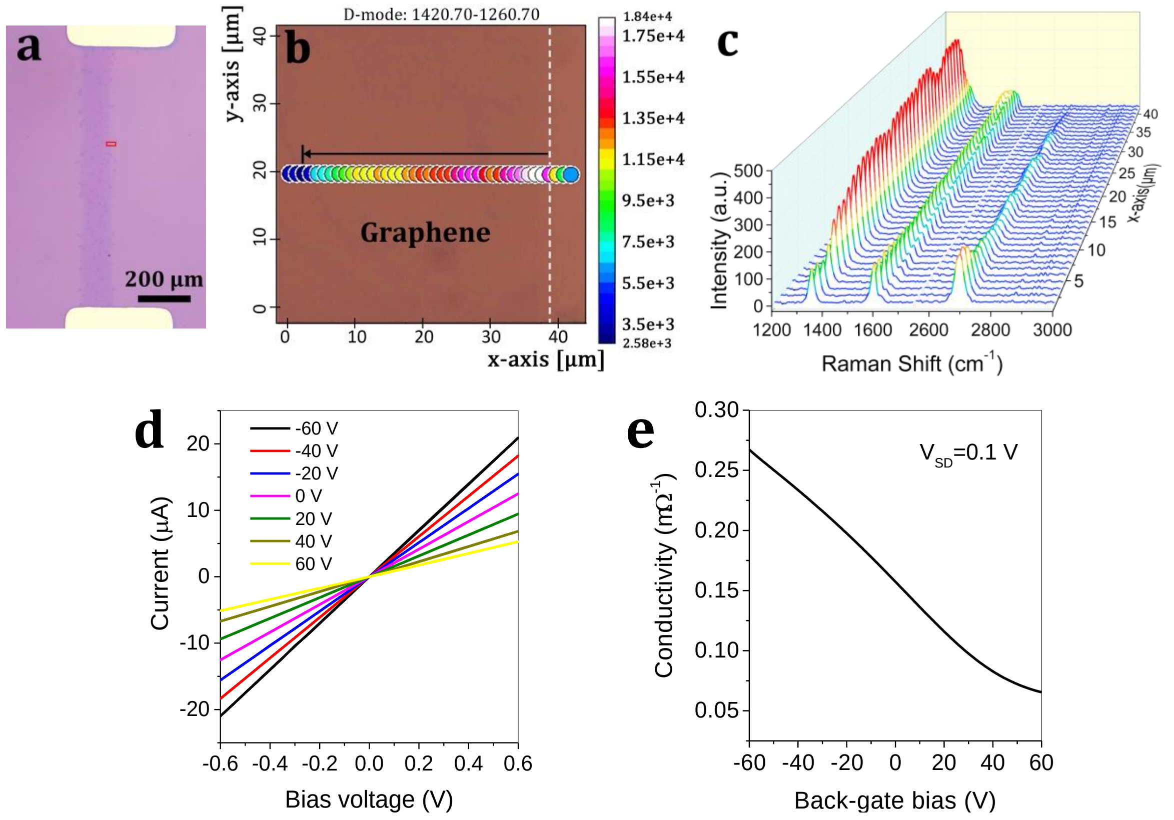

Supplementary Figure 9: Characterization of the graphene FET device (994 µm long and 129 µm wide) patterned by UV ozonation without assistance of a vertical magnetic field. a) Optical images of the shrinking graphene FET device patterned through a 168-µm-wide mask. **b)** Line map of D band crossing one edge of the channel and **c**) the corresponding Raman spectrum evolution. **d)** Linear relationship between source-drain current and voltage for the graphene FET device at different back-gate biases, and **e)** its conductivity as a function of back-gate bias at a fixed source-drain voltage of 0.1 V.

Supplementary Note 1: X-ray photoelectron spectroscopy (XPS) analyses of the UV ozonized graphene under excitation of xenon excimer lamp

XPS is used to determine variation of the chemical functional groups for UV ozonized graphene film under xenon excimer lamp. Freshly exfoliated high quality Kish graphite flakes (~6×6 mm^2) are selected for XPS analyses after the same UV ozonation treatment as that for CVD-grown graphene film[3]. Supplementary Figure 3 shows the survey and highly-resolved XPS spectra for UV ozonized graphite. Compared with the fresh one, an extra O1*s* peak shows up at 532.3 eV (Supplementary Figure 3a), and the highly resolved C1*s* band widens with the full width at half maximum (FWHM) of 0.78 eV (Supplementary Figure 3b). Besides, a new shoulder peak appears at 287.01 eV. The fit of Lorentz curves indicates new emergences of the sp^3 carbon at 284.74 eV and oxygen chemical functional groups of carbonyl (-C=O) at 286.08 eV and epoxide (C-O) at 286.98 eV, respectively[3-5]. The highly resolved O1*s* spectrum (Supplementary Figure 3c) further indicates that the oxygen peak lies at 532.32 eV and its atomic concentration is ~2.29% of the total chemical groups. These results indicate that UV ozonation has a much stronger oxidation capability under irradiation of xenon excimer lamp than that under irradiation of a conventional low-pressure mercury lamp[3].

Supplementary Note 2: Conversion between the volume and molar magnetic susceptibility

Magnetic susceptibility χ is one important parameter to characterize the magnetic properties of a material. A positive susceptibility indicates a paramagnetic material, which will be attracted into the magnetic field. The larger the positive susceptibility, the stronger the attraction force. A negative susceptibility indicates a diamagnetic material, usually with an absolute value of a few orders smaller than that of the paramagnetic material, which will be slightly repelled by the magnetic force.

Both volume magnetic susceptibility (χ_v) and molar magnetic susceptibility (χ_{mol}) have been used to represent the magnetic characteristics of a material in the past. For easy

comparison, we convert the volume magnetic susceptibilities (SI unit) of oxygen and nitrogen gases appeared in the previous work (Supplementary References 1,2) into molar magnetic susceptibilities ($cm^3 \cdot g^{-1}$) taking Supplementary Equation 1.

$$\chi_{mol}=M\chi_v/\rho \qquad \text{(Supplementary Equation 1)}$$

where *M* is the molar mass, and ρ is the density of the material.

According to the ideal gas law *pV=nRT* and the density expression of *ρ=nM/V*, *M* can be expressed as Supplementary Equation 2.

$$M=RT\rho/p \qquad \text{(Supplementary Equation 2)}$$

In the above expressions, *R* is the ideal gas constant, equal to 8.314×10^{-6} $cm^3 \cdot Pa \cdot mol^{-1} \cdot K^{-1}$, *T* is the absolute temperature, *p* is the gas pressure, *n* is the amount of substance or number of moles. Substituting Supplementary Equation 2 into Supplementary Equation 1, we obtain:

$$\chi_{mol}=RT\chi_v/p \qquad \text{(Supplementary Equation 3)}$$

For oxygen and nitrogen gases with a pressure of 1 atm (1.01×10^{-5} Pa) at room temperature (293 K), their volume magnetic susceptibility is 1.447×10^{-7} and -4.91×10^{-10}, respectively, referring to the value given by Hector[1,2]. Substituting these values into Supplementary Equation 3, we obtain the molar magnetic susceptibilities of 3490×10^{-6} $cm^3 \cdot mol^{-1}$ and -11.8×10^{-6} $cm^3 \cdot mol^{-1}$, respectively, for oxygen and nitrogen molecules.

Supplementary Note 3: Patterning graphene through a nickel mask by a horizontal magnetic-field-assisted UV ozonation

Directional motions of oxygen molecules and radicals in an inhomogenous magnetic field are certified by patterning graphene through a nickel mask in Supplementary Figure 5a during UV ozonation. Considering magnetization along *OY* direction for the nickel mask, a stack of bar magnets are used to create an approximately horizontal magnetic field ($\mathrm{B_Y} = 40\ \mathrm{mT}$, $\nabla \mathrm{B_{OY}} = 2\ \mathrm{T \cdot m^{-1}}$) that makes the mask stay stable on graphene sample (see Supplementary Figure 2a,b). Assisted with such a magnetic field in UV ozonation, a completely different

wave-like graphene microstructure is obtained as shown in Supplementary Figure 5b. In this case, all ribs that connect two adjacent holes along *OY* direction disappear. Meanwhile, unsymmetrical wave-like ribs appear with the width of 13.5±1 μm in the narrowest regions. As noticed, the nonsymmetric profile along *OY* direction should be related to specific distribution of the magnetic field[6,7]. Further micro-Raman map of the D band (Supplementary Figure 5c) and the corresponding Raman spectrum evolution (Supplementary Figure 5d) for the outlined wavelike graphene microstructure indicate that defect band appears in the whole surface of graphene due to dissipation of oxidative reactants underneath the mask[3]. The magnetic-assisted UV ozonation has a capability of patterning graphene microstructure with a line width of 13 μm. By properly controlling the horizontal magnetic-field-assisted UV ozonation, it may be a solution to intentionally modifying graphene patterns.

Supplementary References

[1] Wills, A. P. & Hector, L. G. The magnetic susceptibility of oxygen, hydrogen and helium. *Phys. Rev.* **23**, 209-220 (1924).

[2] Hector, L. G. The magnetic susceptibility of helium, neon, argon, and helium. *Phys. Rev.* **24**, 418-425 (1924).

[3] Zhang, Z. et al. Making few-layer graphene photoluminescent by UV ozonation. *Opt. Mater. Express* **6**, 3527-3540 (2016).

[4] Haerle, R., Riedo, E., Pasquarello, A. & Baldereschi, A. sp^2/sp^3 hybridization ratio in amorphous carbon from C1*s* core-level shifts: X-ray photoelectron spectroscopy and first-principles calculation. *Phys. Rev. B* **65**, 045101 (2001).

[5] Yuan, J. *et al*. Tuning the electrical and optical properties of graphene by ozone treatment for patterning monolithic transparent electrodes. *ACS Nano* **7**, 4233-4241 (2013).

[6] Tinkham, M. & Strandberg, M. W. P. Interaction of molecular oxygen with a magnetic field. *Phys. Rev.* **97**, 951-966 (1955).

[7] Ueno, S., Iwasaka, M., Eguchi, H. & Kitajima, T. Dynamic behavior of gas flow in gradient magnetic fields. *IEEE Transactions on Magnetic* **29**, 3264-3266 (1996).
